# MDM4 was associated with poor prognosis and tumor-immune infiltration of cancers

**DOI:** 10.1186/s40001-024-01684-z

**Published:** 2024-01-27

**Authors:** Jie Liu, Jie Yang, Qilong Pan, Xiangyu Wang, Xinyin Wang, Han Chen, Xiaoling Zheng, Qingling Huang

**Affiliations:** 1 Department of Biochemistry and Molecular Biology, School of Basic Medical Sciences, Fujian Medical University, Fuzhou, China; 2 Department of Endoscopy, Shengli Clinical Medical College of Fujian Medical University, Fujian Provincial Hospital, Fuzhou, China; 3 The Graduate School of Fujian Medical University, Fuzhou, China

**Keywords:** MDM4, Pan-cancer, Prognostic biomarker, Tumor-immune infiltration, Inhibitor

## Abstract

MDM4 is one of the MDM protein family and is generally recognized as the key negative regulator of p53. As a cancer-promoting factor, it plays a non-negligible role in tumorigenesis and development. In this article, we analyzed the expression levels of *MDM4* in pan-cancer through multiple databases. We also investigated the correlations between *MDM4* expression and prognostic value, immune features, genetic mutation, and tumor-related pathways. We found that *MDM4* overexpression is often accompanied by adverse clinical features, poor prognosis, oncogenic mutations, tumor-immune infiltration and aberrant activation of oncogenic signaling pathways. We also conducted transcriptomic sequencing to investigate the effect of MDM4 on transcript levels in colon cancer and performed qPCR to verify this. Finally, we carried out some in vitro experiments including colony formation assay, chemoresistance and senescence-associated β-galactosidase activity assay to study the anti-tumor treatment effect of small molecule MDM4 inhibitor, NSC146109. Our research confirmed that MDM4 is a prognostic biomarker and potential therapeutic target for a variety of malignancies.

**Supplementary Information:**

The online version contains supplementary material available at 10.1186/s40001-024-01684-z.

## Introduction

Cancer is the leading cause of death throughout the world according to estimations from the World Health Organization (WHO), which imposes a substantial health and economic burden because of its high mortality [1]. Traditional treatments like surgery, chemotherapy or radiation therapy, prolong survival in patients with tumors, and encounter stringent bottlenecks simultaneously. For several years now, molecular-targeted therapy and immunotherapy have become promising approaches to own higher response rates and better prognosis in malignancies treatment. Discovering novel tumor biomarkers for early detection and widening the potential therapy options have substantial societal and medical impacts.

The MDM gene family, called mouse double minute because of the isolation from small extrachromosomal bodies present in the mouse cell line, comprises MDM2 and MDM4 [2]. MDM2, first identified in 1992, has been elucidated to stimulate tumors’ development in multiple cancers through activating the ubiquitination of p53 mediated by the proteasome. Mouse Double Minute 4 (*MDM4*), also known as *MDMX*, has been mapped to chromosomal 1q32, the encoded protein of which consists of 490 amino acids. As one of MDM family members, MDM4 has similar activities to MDM2 and also plays a negative regulatory role in p53 levels [3]. The N-terminal of MDM4 is a p53 binding domain that can bind with the transcriptional activation domain to inhibit p53 from activating its target genes. During the connection procedure, the N-terminal of p53 projects three hydrophobic residues (phe19, trp23, and leu26) into the hydrophobic cleft on the surface of MDM4 [4]. The central acidic domain and zinc-finger domain contribute to protein folding and functional regulation. The MDM4 C terminus comprises a RING domain in combination with MDM2 and promotes its regulation of p53 [5].

The impact of MDM4 on tumor development also depends on its alternative splicing, including the two most studied isoforms, MDM4-FL (full-length) and MDM4-SF (short form). MDM4-FL exhibits stronger protein stability, while the high expression of MDM4-S is ubiquitous in tumor cells [6, 7]. MDM4 isoforms are also closely related to tumor progression. For example, high expression of MDM4-A (one of MDM4 isoforms) in human melanoma samples is correlated with poor prognosis [8], while high MDM4-ALT2 expression in rhabdomyosarcoma likely foreshadows an increased risk of metastasis [9]. Although extensive research has been carried out on the carcinogenic effects of MDM4, the role of MDM4 in pan-cancer is still elusive.

In this research, we analyzed the correlations between *MDM4* expression and prognostic value, immune features, genetic mutation, or tumor related pathways in pan-cancer through multiple databases. Furthermore, we explored the anti-tumor efficacy of small molecule MDM4 inhibitor in vitro experiments. In brief, our studies shows that MDM4 is emerging as an attractive therapeutic target for a variety of malignant neoplasms.

## Materials and methods

### Gene expression analysis

From the UCSC database (https://xenabrowser.net/datapages/), we downloaded the processed TCGA pan-cancer RNA-seq data as normalized transcripts per million (TPM) value which was further transformed to log_2_(TPM + 1). R (version 3.6.4) software was applied to calculate the gene expression levels, and the Wilcoxon Rank Sum and Signed Rank Tests were applied for significant difference analysis. The SangerBox website [10] is an online platform which was used for analysis and visualization. In addition, we also obtained the mRNA expression data of cell lines from the Cancer Cell Line Encyclopedia database (https://depmap.org/portal/gene/MDM4?tab=overview), and the protein expression data of MDM4 from the Human Protein Atlas (https://www.proteinatlas.org/ENSG00000198625-MDM4/pathology). Statistical results were analyzed using GraphPad Prism.

### Clinical data analysis

Statistical analysis was sequentially performed for the integrated TCGA Pan-Cancer clinical data, including clinicopathologic variables, serum biochemical parameters and follow-up information [11]. For more than two groupings, *p* values were corrected for Dunn’s multiple comparisons test using the Bonferroni approach. The differences reaching statistical significance in the results were visualized by the R package “pROC” (version 1.17.0.1) and “ggplot2” (version 3.3.3). Using the R package “survminer” (version 0.4.9) and “survival” (version 3.2), Kaplan–Meier (KM) survival curves of overall survival (OS), disease-specific survival (DFS) and progress-free interval (PFI) were plotted by the minimum *p* value (Cox regression analysis) approach.

### Mutation and immune infiltration analysis

Simple nucleotide variation (SNV) datasets generated by Mutect2 and copy number variation (CNV) datasets generated by GISTIC were downloaded. Functions in the R package “maftools” (version 2.8.05) [12] were called to calculate the tumor mutation burden (TMB), homologous recombination deficiency (HRD), loss of heterozygosity (LOH), mutant-allele tumor heterogeneity (MATH), microsatellite instability (MSI), and Neoantigens. The Spearman's correlation coefficients were presented as radar charts by the R package “ggradar” (version 0.2). Kruskal–Wallis rank sum test was performed to establish the significance of differences among the CNV neutral-, gain- or loss- groups.

Heatmap of immune cell-infiltration abundance was done utilizing different mRNA-based immune infiltration prediction algorithms such as TIMER [13], MCPCounter [14], EPIC [15], ssGSEA, and ESTIMATE [16]. “Corr. test” function in R package “psych” (version 2.1.6) was used to calculate the Pearson’s correlation coefficient between the *MDM4* mRNA expression level and the degree of immune cell infiltration.

### Functional annotation and enrichment analysis

We performed the clusterProfiler (version 4.2.0) R package [17] to Gene Ontology (GO), Kyoto Encyclopedia of Genes and Genomes (KEGG) [18–20], and Gene Set Enrichment Analysis (GSEA) for the differentially expressed genes (DEGs). The enrichment results and volcano plot were visualized by “ggplot2” (version 3.3.3). Adjusted *p* value < 0.05 was considered as significant.

### Cell culture and viability assay

Human cell lines HCT116, 786-O and A549 were purchased from Hunan Fenghui Biotechnology Co., Ltd. (Changsha, China). Cell lines SW480, MCF-7 and Huh7 were purchased from National Collection of Authenticated Cell Cultures (Shanghai, China). THP-1 was purchased from Fuzhou Zolgene Biotechnology Co., Ltd. (Fuzhou, China). HCT116 and 786-O were cultured in RPMI1640, A549 in DMEM/F12, SW480, MCF-7 and Huh7 in DMEM. THP-1 was cultured in RPMI1640 with 0.05 mM β-mercaptoethanol. These media were supplemented with 10% FBS, 1% antibiotics, and cells maintained in a 37 ℃ incubator with 5% CO_2_. NSC146109 (HY-108638, MedChemExpress, USA) and Oxaliplatin (OXA) were dissolved in DMSO at a final concentration of 10 mM and stored at − 20 ℃. The stock was diluted to the required concentration with corresponding medium when needed.

Cells were seeded in a 96-well plate with 8000 cells per well. The next day, cells were treated with various concentration of NSC146109 or OXA for 48 h. Before measuring the OD value in 450 nm, 10% CCK8 was added and cells were cultured in a 37 ℃ incubator for 1 h.

### Colony formation assay

Cells were seeded in 6-well plates with 1000 cells per well for 14 days. Experimental groups were treated with NSC146109 at a concentration of 0.5 μM, and the control group was treated with the same concentration of DMSO. Then cells were fixed in methanol for 1 min, stained with 0.5% crystal violet at room temperature for 5 min and carefully rinsed with tap water.

### Quantitative real-time PCR

Total RNA was extracted using TRIzol reagent and reversely transcribed to cDNA using a Takara cDNA Synthesis Kit according to the manufacturer’s instructions. Quantitative real-time PCR was performed with SYBR Green incorporation. The primer sequences are listed in Additional file 1: Table S1.

### Western blot

Cells were seeded in 6-well plates equally. On the next day, 0.5 μM NSC146109 was added in experimental groups and equal amount of DMSO was added in the control groups. After incubation for 24 h, cells were lysed with RIPA lysis buffer with added protease (1:100 dilution; MilliporeSigma) inhibitors. Protein concentrations were measured by the Beyotime BCA Protein Assay Kit (P0012, Beyotime). Protein extracts were separated by SDS-PAGE, transferred onto polyvinylidene fluoride (PVDF) membranes (P2938, Sigma-Aldrich), and blocked in 5% milk in TBST. Then the PVDF membranes were blotted individually with MDM4 (A300-287A, Bethyl Laboratories, Germany) and β-actin (23660-1-AP, proteintech, Germany) antibodies.

### Senescence-associated (SA) β-galactosidase activity assay

Cells were plated in six-well plates at a seeding density of 1 × 10^6^ cells per well. After attachment to the wall, the cells were treated with 1 μM NSC146109 or DMSO. According to the instruction of micro β-galactosidase (β-gal) assay kit (Solarbio, Beijing, China), cells were then washed with PBS, fixed with 2% formaldehyde/0.2% glutaraldehyde for 10 min, and stained with SA-β gal solution at 37 °C overnight. Leica DMi8 microscope and LAS X Core software was used to take and process images.

### Transcriptomic sequencing

Total RNA is first extracted from the sample, and mRNA is enriched using magnetic beads with Oligo (dT). The mRNA is fragmented using a Fragmentation Buffer, and then the first strand of cDNA is synthesized using six base random primers. Buffer, dNTPs, RNase H, and DNA polymerase I are added to synthesize the second strand of cDNA. Next, the resulting cDNA is then purified using a QiaQuick PCR kit and eluted with EB buffer. End-repair is done and base A is added to the cDNA fragments, followed by sequencing connector ligation. The target size fragments are recovered by agarose gel electrophoresis, and PCR amplification is carried out to complete the preparation of the entire library. Finally, the constructed library is sequenced using the Illumina HiSeq2000 platform to obtain high-throughput sequencing data that can be used for downstream analysis. The RNA-seq data from this study have been disclosed in the GEO database GSE243108.

### Migration experiment

Stable expressing GFP and MDM4 colon cancer cells SW480 were evenly seeded in a 24-well plate. An upper chamber of a Transwell insert was coated with 60,000 THP-1 and placed in a CO_2_ incubator for cultivation. After 48 h, the Transwell insert was removed and washed three times with 1xPBS. It was then stained with 500 μL of crystal violet for 10 min, followed by three washes with 1xPBS buffer. After gently wiping and air-drying, photographs were taken under a microscope.

### Immunohistochemistry

Prepared slices were placed in a preheated constant-temperature incubator at 65 °C for 2 h, followed by dewaxing and hydration. Citrate buffer boiling method (0.01 M PBS, pH 6.0) was used to perform antigen retrieval. After three washed with PBST, endogenous peroxidase blockers were added and incubated at room temperature for 10 min. The primary antibody (MDM4, 1:500) was added at 4 °C overnight. After overnight incubation, a reaction enhancer was sequentially added and incubated at room temperature for 12 min, followed by the addition of a universal HRP secondary antibody and incubation at room temperature for 30 min. DAB staining was performed for 1.5 min, followed by rinsing with tap water. Counterstaining with hematoxylin was done for 35 s, and rinsing was done with flowing water after ammonia water bluing. The slices were then dehydrated, cleared, and finally mounted with neutral mounting medium. Following the guidelines of Helsinki Declaration, the studies involving human participants were reviewed and approved by the Ethics Committee of Fujian Medical University (2021-FJMU-015), and written informed consent was obtained from each participant.

## Results

### Expression levels of MDM4 across different tumor types

**Fig. 1:**
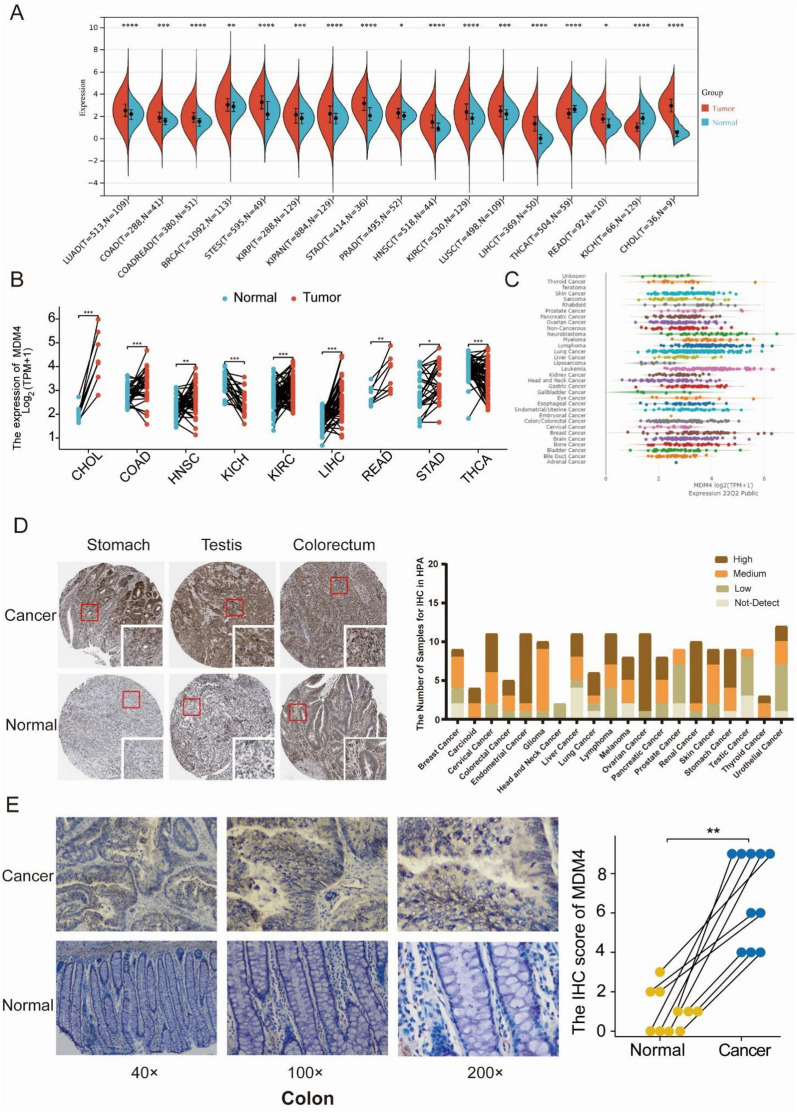
The expression levels of *MDM4* in different cancer types. **A** The differentially expressed *MDM4* between normal and tumor samples in TCGA pan-cancer. **B** Comparisons of *MDM4* expression levels in TCGA tumor samples and paired normal controls. **C** The expression levels of *MDM4* in different cell lines using the Cancer Cell Line Encyclopedia database. **D** Histograms of the MDM4 protein expression levels and examples of Immunohistochemistry images from the Human Protein Atlas. **E** Representative images from immunohistochemical staining of 10 cases of colon cancer tissues and its corresponding adjacent tissues for MDM4 and their IHC scores. ns, *p* ≥ 0.05; *, *p* < 0.05; **, *p* < 0.01; ***, *p* < 0.001

Through a TCGA pan-cancer analysis, the *MDM4* mRNA expression levels were initially watched. As documented in Fig. 1A, *MDM4* was significantly overexpressed in 15 common cancers: Lung adenocarcinoma (LUAD), Colon adenocarcinoma (COAD), Rectum adenocarcinoma (READ), Breast invasive carcinoma (BRCA), Stomach and Esophageal carcinoma (STES), Kidney renal papillary cell carcinoma (KIRP), Pan-kidney cohort (KIPAN), Stomach adenocarcinoma (STAD), Prostate adenocarcinoma (PRAD), Head and Neck squamous cell carcinoma (HNSC), Kidney renal clear cell carcinoma (KIRC), Lung squamous cell carcinoma (LUSC), Liver hepatocellular carcinoma (LIHC) and Cholangiocarcinoma (CHOL). For the paired tumor and adjacent normal specimens (Fig. 1B), the *MDM4* expression of tumor tissue in 7 cancer types was also significantly higher than that in its corresponding adjacent tissues, including BRCA, CHOL, COAD, Esophageal carcinoma (ESCA), HNSC, LIHC, and STAD. Meanwhile, it was significantly downregulated in Thyroid carcinoma (THCA) and Kidney chromophobe (KICH) in both paired and unpaired analyses. These elevated expression levels were confirmed reciprocally in the cancerous cell lines (Fig. 1C). In addition, we investigated the localization and expression of *MDM4* at the protein level using the Human Protein Atlas (HPA) database. Moderate to strong cytoplasmic and nuclear positivity were observed in most cancer tissues, especially in endometrial cancer, ovarian cancer and renal cancer (Fig. 1D). Representative immunohistochemistry images of MDM4 protein in tumor samples and its corresponding adjacent tissues are described in Fig. 1D. We additionally we obtained 10 cases of human colon cancer tissues and performed immunohistochemical analysis (Fig. 1E). As expected, the expression of MDM4 in tumor samples was higher than that in paired normal tissues.

### The clinical relevance and prognostic value of MDM4 expression

**Fig. 2:**
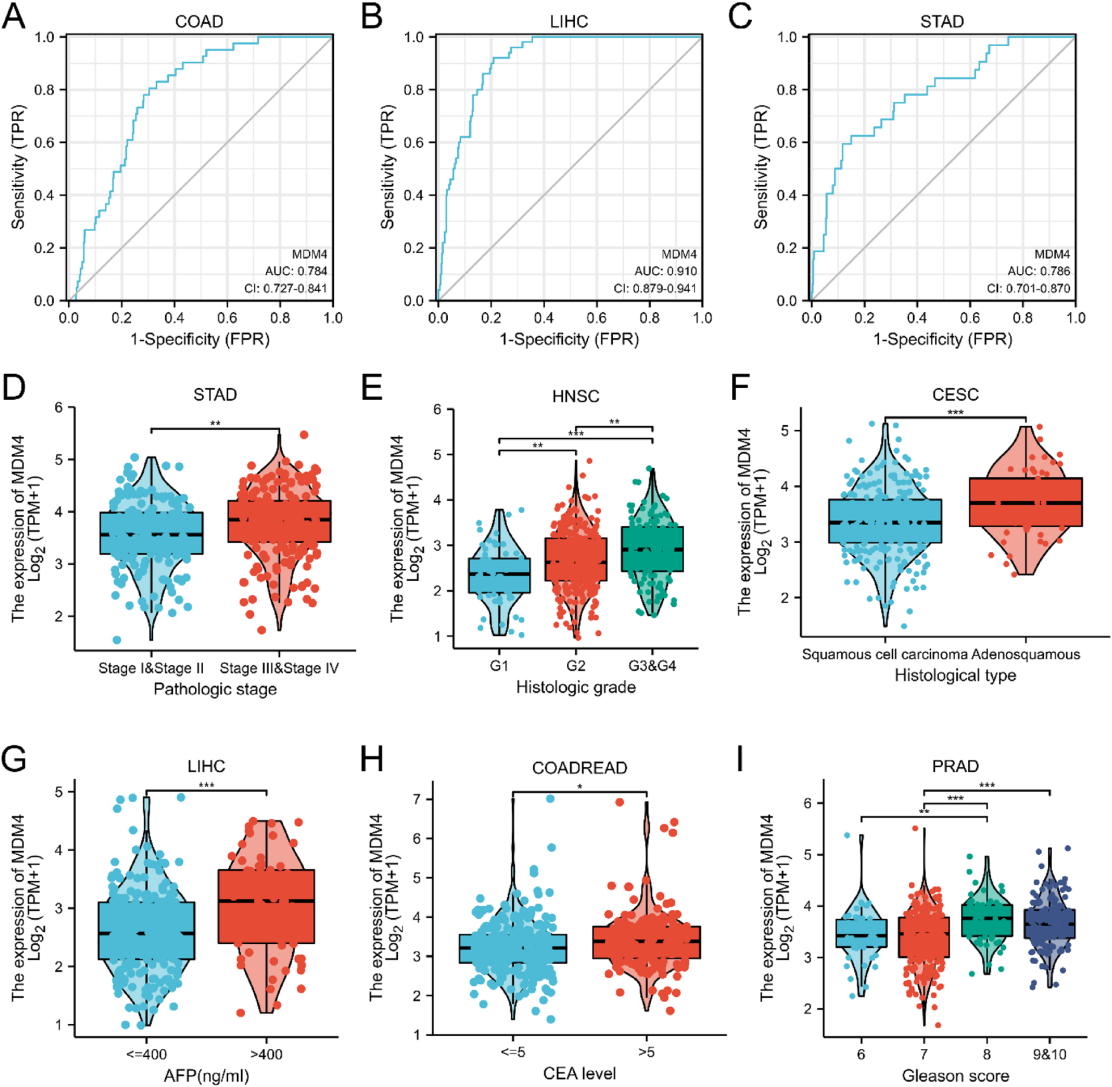
The correlation between the clinical features and *MDM4* expression. **A, B** and **C** Receiver Operating Characteristic Curve analysis for the expression levels of *MDM4* in COAD, LIHC and STAD. Correlation between the *MDM4* expression and pathological stages of STAD **D**, histologic grading of HNSC **E**, histological type of CESC **F**, serum AFP concentration in LIHC **G**, serum CEA level in COADREAD **H**, or Gleason score of PRAD **I**. TPR, True-Positive Rate; FPR, False-Positive Rate; AUC, Area under the Curve; AFP, alpha fetoprotein; CEA, carcinoembryonic antigen

First, we explored the diagnostic value of *MDM4* for the malignant cancers. After calculating the area under the ROC curves (AUC), it was found that *MDM4* has good diagnostic performances in COAD (AUC = 0.784), LIHC (AUC = 0.910), STAD (AUC = 0.786), HNSC (AUC = 0.721), KICH (AUC = 0.938), KIRC (AUC = 0.783), and READ (AUC = 0.804) (Fig. 2A–C, Additional file 1: Fig S1A–D). Second, we examined the correlation between the clinical variables and *MDM4* expression. In STAD and HNSC, high *MDM4* expression was associated with late clinical stage (Fig. 2D, Additional file 1: Fig. S1E), and it was contrary to that in THCA (Additional file 1: Fig. S1F). Histologically, the high-level expression of *MDM4* correlates with worse differentiation in HNSC, Glioma (GBMLGG) and LIHC (Fig. 2E, Additional file 1: Fig. S1G-H). In cervical squamous cell carcinoma and endocervical adenocarcinoma (CESC) and ESCA, adenocarcinoma had a higher *MDM4* mRNA expression compared to the squamous cell carcinoma (Fig. 2F, Additional file 1: Fig. S1I). Moreover, the upregulated expression of *MDM4* was often accompanied by adverse clinical features, such as AFP > 400 ng/ml in LIHC, CEA > 5 ng/ml in COADREAD, and Gleason score > 8 in PRAD (Fig. 2G–I).

**Fig. 3:**
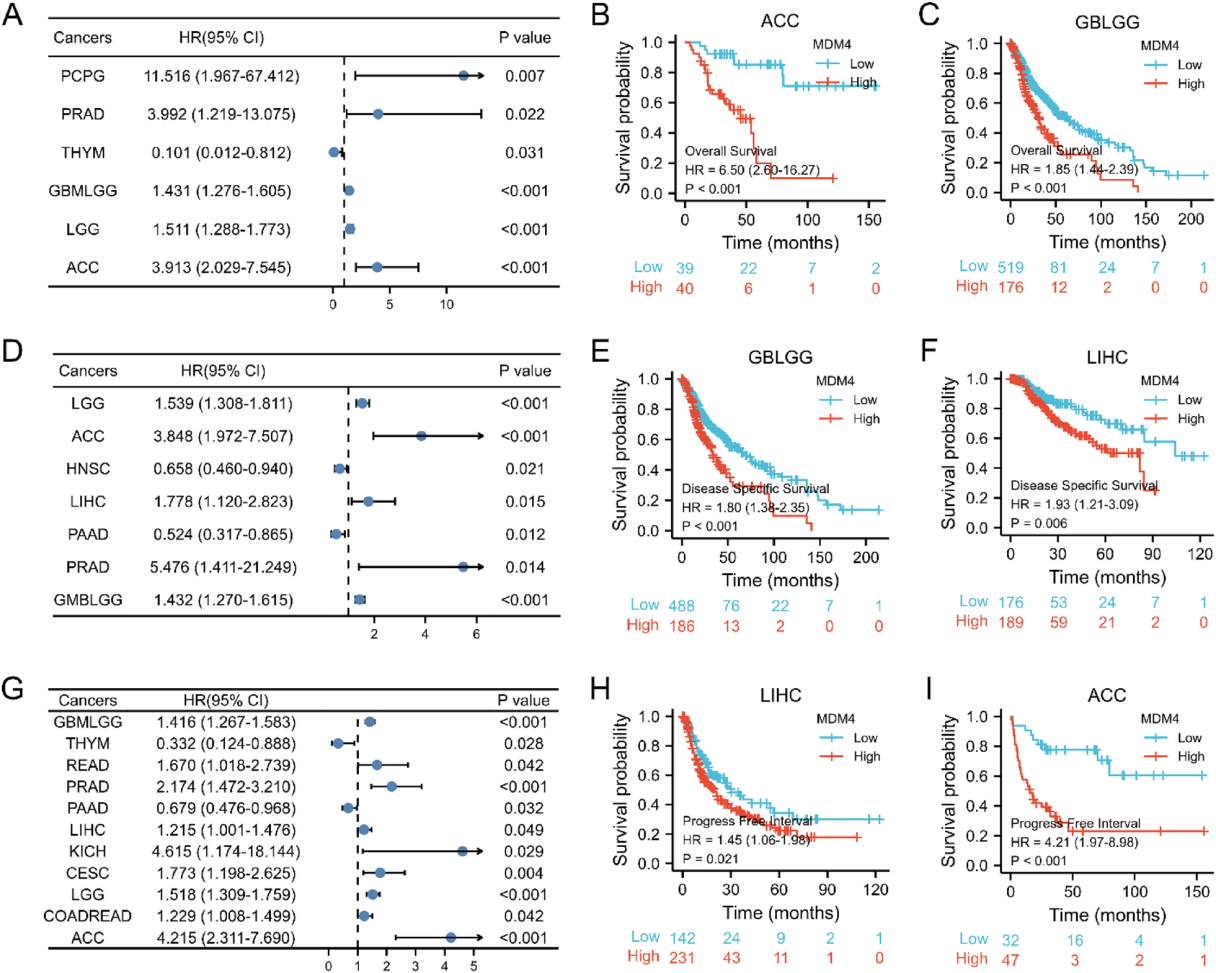
Correlations between *MDM4* expression and prognostic value. **A** Forest plot of hazard ratios for overall survival. **B** and** C** Kaplan–Meier survival curves for overall survival in ACC and GBLGG. **D** Forest plot of hazard ratios for disease-specific survival. **E** and** F** Kaplan–Meier survival curves for disease-specific survival in GBLGG and LIHC. **G** Forest plot of hazard ratios for progress-free interval. **H** and** I** Kaplan–Meier survival curves for progress-free interval in LIHC and ACC. HR, hazard ratio; CI, confidence interval

Next, the significant results on univariate Cox regression analysis (*p* ≤ 0.05) for OS are presented in Fig. 3A and Additional file 1: Fig. S2. *MDM4* was displayed to be of prognostic value for Pheochromocytoma and paraganglioma (PCPG) (HR = 11.516, *p* = 0.007), PRAD (HR = 3.992, *p* = 0.022), Thymoma (THYM) (HR = 0.101, *p* = 0.031), GBMLGG (HR = 1.431, *p* < 0.001), Brain lower grade glioma (LGG) (HR = 1.511, *p* < 0.001), and Adrenocortical carcinoma (ACC) (HR = 3.913, *p* < 0.001). The representative K–M plotter for ACC and GBMLGG are described in Fig. 3B–C. Then, the Cox proportional hazards were determined likewise for the DSS: ACC (HR = 3.848, *p* < 0.001), LGG (HR = 1.539, *p* < 0.001), HNSC (HR = 0.658, *p* = 0.021), LIHC (HR = 1.778, *p* = 0.015), pancreatic adenocarcinoma (PAAD) (HR = 0.524, *p* = 0.012), PRAD (HR = 5.476, *p* = 0.014), and GBMLGG (HR = 1.432, *p* < 0.001) (Fig. 3D–F). Finally, *MDM4* was identified as the prognostic factors for PFI in various cancer types: GBMLGG (HR = 1.416, *p* < 0.001), THYM (HR = 0.332, *p* < 0.028), READ (HR = 1.670, *p* < 0.042), PRAD (HR = 2.174, *p* < 0.001), PAAD (HR = 0.679, *p* = 0.032), LIHC (HR = 1.215, *p* = 0.049), KICH (HR = 4.615, *p* = 0.029), CESC (HR = 1.773, *p* = 0.004), LGG (HR = 1.518, *p* < 0.001), COADREAD (HR = 1.229, *p* = 0.042), and ACC (HR = 4.215, *p* < 0.001) (Fig. 3G–I). Collectively, *MDM4* can be proposed as a novel biological marker to provide the characterization of high-risk tumors.

### Genomic alteration analysis 

**Fig. 4:**
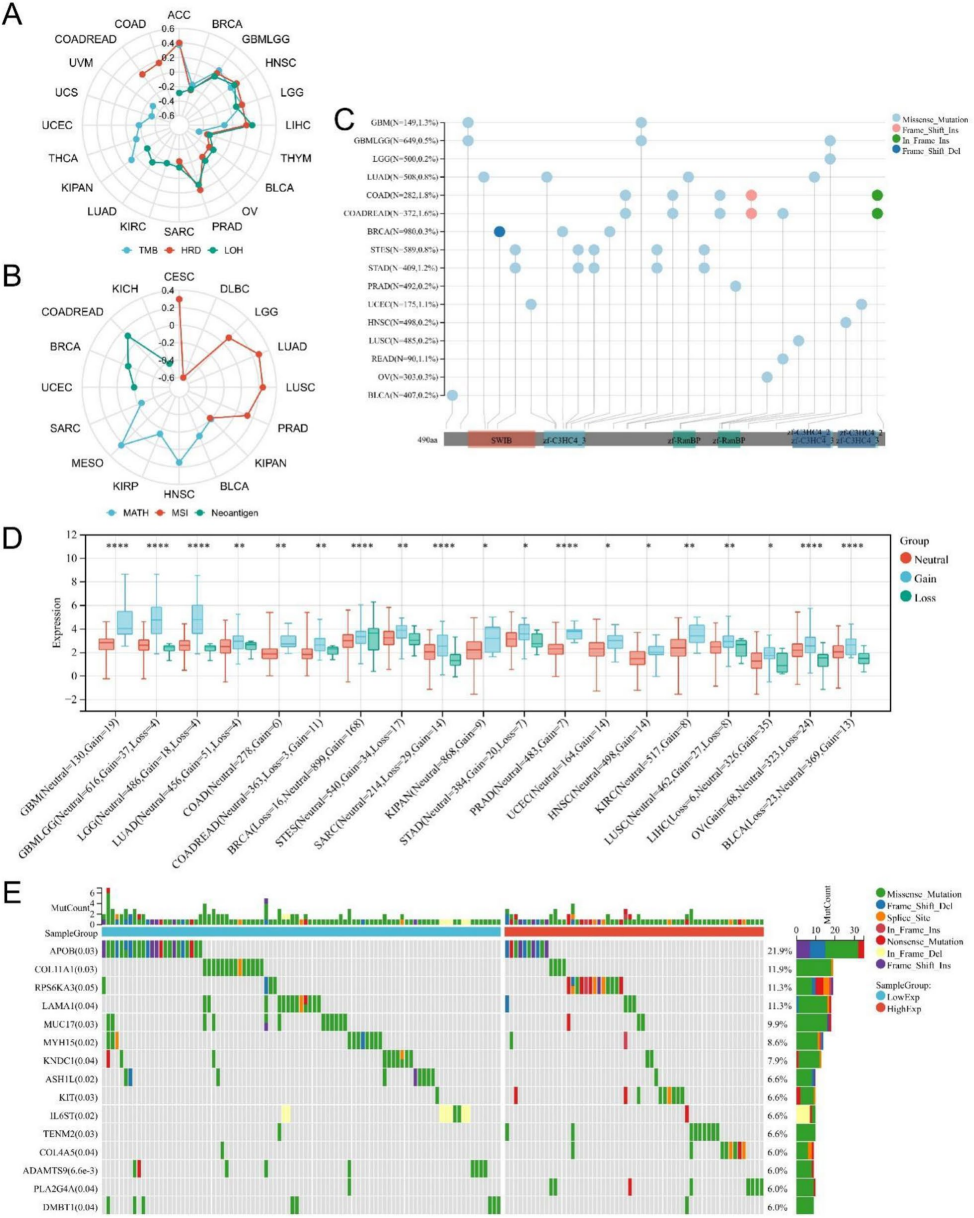
Genomic alteration analysis of *MDM4*. **A** and **B** Radar charts representing Spearman's correlation coefficients between *MDM4* expression and TMB, HRD, LOH, MATH, MSI or Neoantigen. **C** Overview of the mutational frequency across different tumor types. **D** Copy number variation analysis of *MDM4* expression. **E** Mutation waterfall plot between the high vs. low *MDM4* expression groups. TMB, tumor mutation burden; HRD, homologous recombination deficiency; LOH, loss of heterozygosity; MATH, mutant-allele tumor heterogeneity; MSI, microsatellite instability; DEL, deletion; INS, insertion

To explore the role of *MDM4* in tumorigenesis, malignant progression and tumor lethality, we evaluated the genomic heterogeneity index, which is known to affect clinical outcomes such as TMB, HRD, LOH, MATH, MSI, and Neoantigens. Only the major cancer types with significant correlations (*p* < 0.05) were highlighted in the radar charts (Fig. 4A–B). In ACC, LIHC and PRAD, high *MDM4* expression was concomitant with high TMB, HRD and LOH, implying less efficacious chemotherapy and immunotherapy. In CESC, LUAD and LUSC, *MDM4* expression correlated positively with MSI resulting in DNA mismatch repair deficiency. However, this might be unrelated to its own gene mutations which were mainly dominated by missense mutations (mutation rate: 0.2–1.8%) except for the frame-shift mutations in COADREAD (mutation rate = 1.8%) (Fig. 4C). We next focused on the association between *MDM4* expression and CNVs because it is well-known that CNV-high tumors have greater malignant potential. Figure 4D shows that *MDM4* was highly expressed in pan-cancer samples with CNV gains. Although the data cannot provide an explanation for the causality, constructed mutational landscape reflected disparate mutation frequency between different *MDM4* expression groups in LIHC, COAD, STAD and PRAD (Fig. 4E, Additional file 1: Fig. S3). In high *MDM4* expression group of LIHC, oncogenes (*APOB*, *COL11A1*, and *LAMA1*) had fewer mutations and low-frequency mutated genes (*RPS6KA3*, *KIT*, *COL4A5*) were more prone to mutations. These results indicates that *MDM4* was involved in tumor-promoting mutations.

### Immune cell infiltration 

**Fig. 5:**
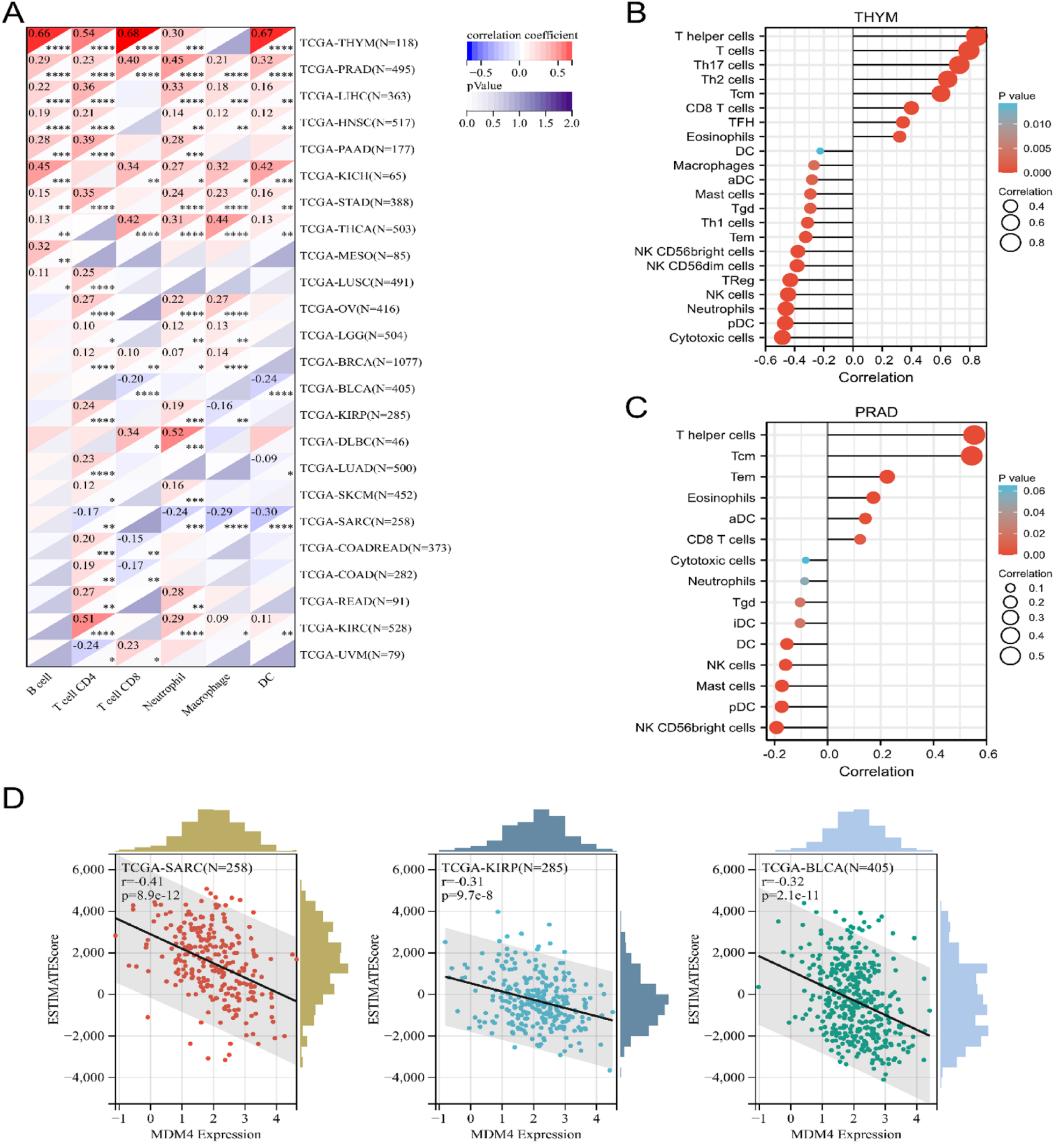
Correlation of *MDM4* expression with immune infiltration in different cancers. **A** Heat map of Pearson's correlations displaying the level of immune cell infiltration using the TIMER algorithm. **B** and **C** Lollipop plots of Spearman's correlations between *MDM4* expression and infiltrating immune cells in THYM and PRAD using the ssGSEA algorithm. **D** Pearson’s correlation analysis for ESTIMATE score in SARC, KIRP and BLCA. SARC, sarcoma; DC, dendritic cell; aDC, activated DC; iDC, immature DC; pDC, plasmacytoid DC; Tcm, T central memory; Tgd, T gamma delta; Tem, T effector memory; TFH, T follicular helper

Multiple algorithms were used for the evaluation of infiltrating immune microenvironment. Results from TIMER database reveals that *MDM4* is positively correlated to multiple different infiltrating immune cells in THYM, KICH and THCA (Fig. 5A). This is consistent with the protective factors above. In most cases, however, *MDM4* expression correlated positively with T cells but negatively with the degree of major histocompatibility complex (MHC), macrophages and natural killer (NK) cells infiltration (Additional file 1: Fig. S4). In specific, it contains CD4 + T cells, CD8 + T cells, T helper cells and central memory T cells (Fig. 5B–C). These should be considered as inflammatory cell infiltration rather than anti-tumor immune response because the ESTIMATEScore has a linear decline in diverse kind malignant tumors with increasing *MDM4* expression (Fig. 5D**)**. In other words, future treatments targeting *MDM4* might enhance anti-tumor immunity.

### Functional enrichment analysis of MDM4-related genes

**Fig. 6:**
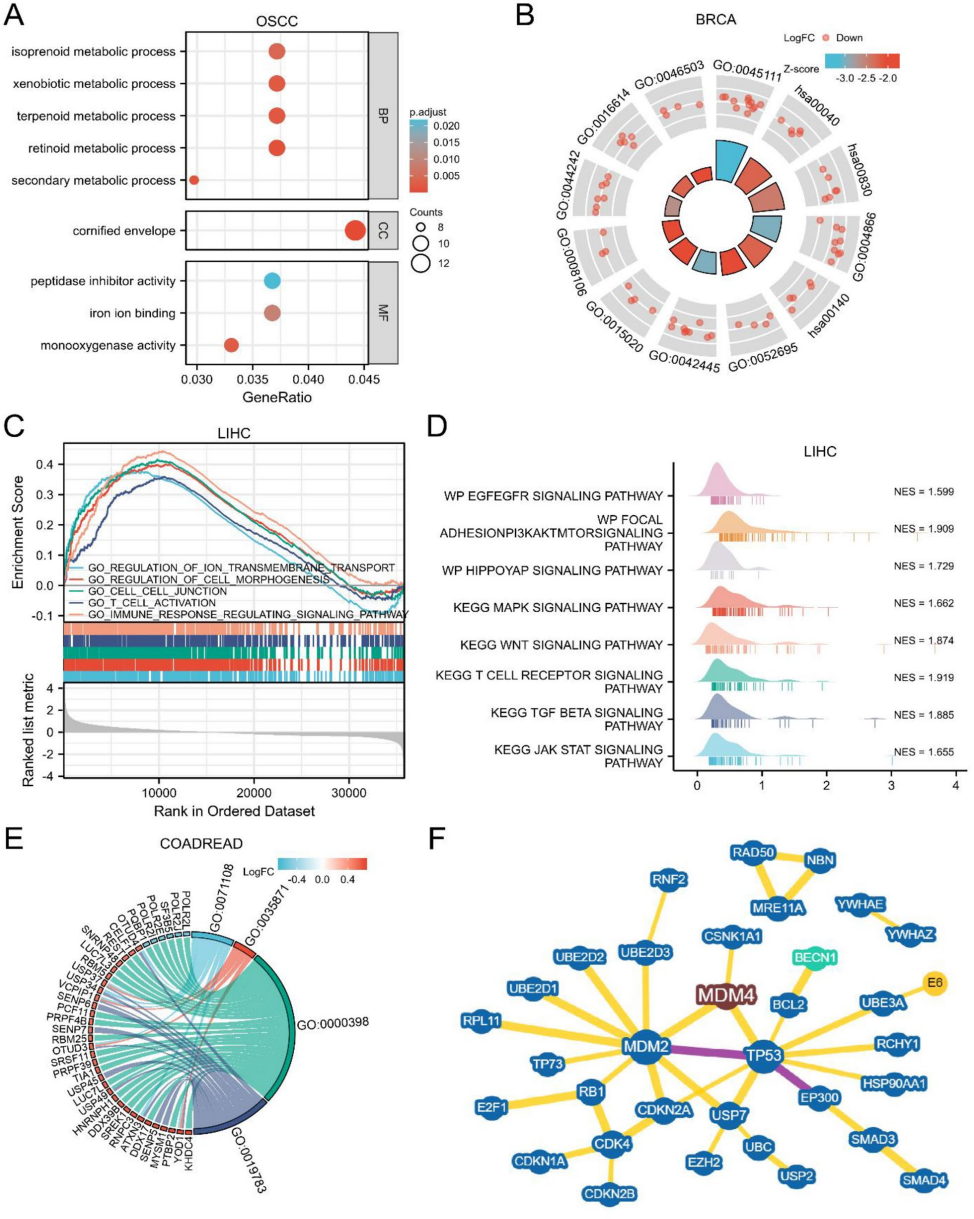
Functional enrichment analysis of *MDM4*-related genes. **A** GO enrichment of differentially expressed genes in OSCC. **B** GO enrichment of differentially expressed genes with logFC values in BRCA. **C** and **D** Relevant signaling pathways of *MDM4*'s GSEA in LIHC. **E** GO enrichment of co-expressed genes in COADREAD; **F** prediction of *MDM4*-substrate relationships based on BioGRID database. OSCC, oral squamous cell carcinoma; BP, biological process; CC, cellular component; MF, molecular function; NES, normalized enrichment scores

We tried to explore the potential mechanism of *MDM4* at molecular-level through related gene enrichment analysis from multiple viewpoints. Patients were sorted into high- or low-expression groups according to the expression of *MDM4*, and DEGs analysis was performed. By GO and KEGG functional enrichment analysis of DEGs, we found that in OSCC (oral squamous cell carcinoma) *MDM4* is closely related to secondary metabolic process, cornified envelope and iron ion binding (Fig. 6A). Once the logFC values are considered, however, in BRCA the negative correlations are observed between *MDM4* and tumor cell metabolism such as cellular glucuronidation (GO:0052695), lipid catabolic process (GO:0044242), and alcohol dehydrogenase (NADP+) activity (GO:0008106) (Fig. 6B). In LIHC, GSEA was performed to define the pathway functional annotation between *MDM4* high- versus low-expressing tumors. *MDM4* has significant impacts on cell junction, immune response, regulation of cell morphogenesis, ion transmembrane transport, and multiple oncogenic signaling pathways, as shown in Fig. 6C–D. Another approach is to investigate the co-expressed molecules when studying the functions of *MDM4*. We screened the co-expressed genes with correlation coefficient greater than 0.5 in COADREAD, and the GO functional enrichment shows that they might affect the protein K48-linked deubiquitination (GO:0071108) and ubiquitin-like protein-specific protease activity (GO:0019783) (Fig. 6E). As an E3 ubiquitin ligase, MDM4 protein can exacerbate CSNK1A1 ubiquitination and degradation, or recognize indirect substrates by the regulation of p53 protein (Fig. 6F). On the whole, the pro-tumor function of *MDM4* is complicated and multifaceted.

### RNA-sequencing indicating immune response when MDM4 is overexpressed

**Fig. 7:**
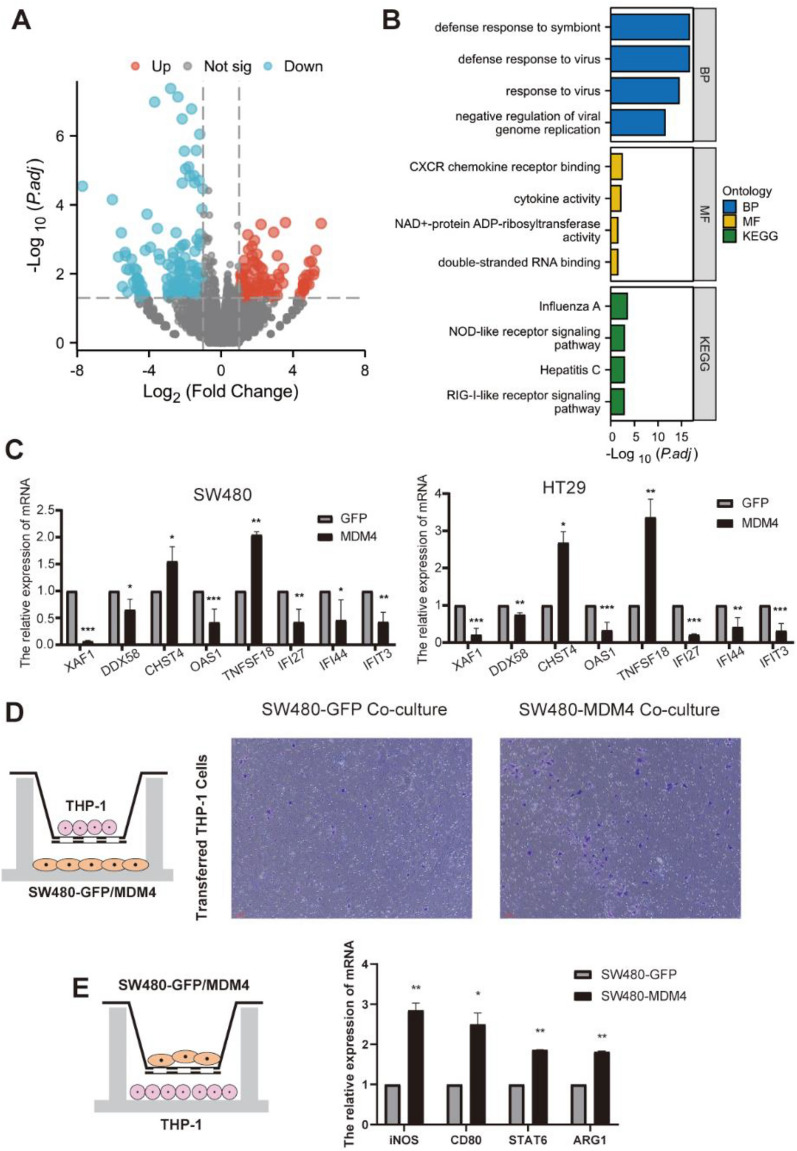
RNA-sequencing indicating immune response when MDM4 is overexpressed. **A** Volcano plot showing differential gene expression in SW480 cells. **B** GOCC: immunoglobulin complex. **C** qPCR validation of selected genes in SW480 and HT29 cells. **D** Migration of THP1 after co-cultured with stable expressing GFP and MDM4 colon cancer cells SW480. **E** The relative mRNA expression in THP1 which was co-cultured with stable expressing GFP and MDM4 colon cancer cells SW480. * *p* < 0.05; **p < 0.01

To explore how MDM4 functions independently of p53, we constructed the p53 mutant cell line overexpressing *MDM4* and analyzed the transcriptomics by RNA sequencing. The colon cancer cell line, SW480 cells, were transiently transfected with plasmids encoding MDM4 protein. The transcriptomics results suggested that there were 227 DEGs (including 98 genes up-regulated and 129 down-regulated genes) when *MDM4* was overexpressed (Fig. 7A). The results of GO and KEGG pathway analyzed for these differentially expressed genes were presented in Fig. 7B. In the p53-mutant colon cancer cell, overexpressed *MDM4* activated immune response-related functional pathways such as response to type I interferon, cytokine receptor binding, chemokine and NF-κB signaling pathway. We selected eight representative immune-related molecules for qPCR validation in SW480 and another colon cancer cell line HT29: *DDX58*, *CHST4, TNFSF18*, *XAF1, OAS1, IFI27, IFI44, IFIT3* (Fig. 7C). *DDX58, XAF1, OAS1, IFI27, IFI44* or *IFIT3* was indeed downregulated and *CHST4* or *TNFSF18* was upregulated in *MDM4* overexpressed colon cancer cells. These results again demonstrated an overactivation of inflammatory response likely caused by MDM4. Because of the close relationship about MDM4 and immunoregulation, we co-cultured the stable transgenic strain SW480 expressing GFP and MDM4 with macrophages THP-1 (SW480 was speeded in transwell upper chambers and macrophages were speeded in transwell lower chamber) to evaluate whether MDM4 affected the migration ability of THP-1 (Fig. 7D). The results showed that MDM4 could promote macrophage migration. To further validate the relationship between MDM4 and immunoregulation, we co-cultured macrophages with SW480 stably expressing GFP and MDM4 (SW480 was speeded in transwell upper chambers and macrophages were speeded in transwell lower chamber), and found that MDM4 might promote M2 polarization of macrophages (Fig. 7E).

### Antitumor efficacy of small molecule MDM4 inhibitor

**Fig. 8:**
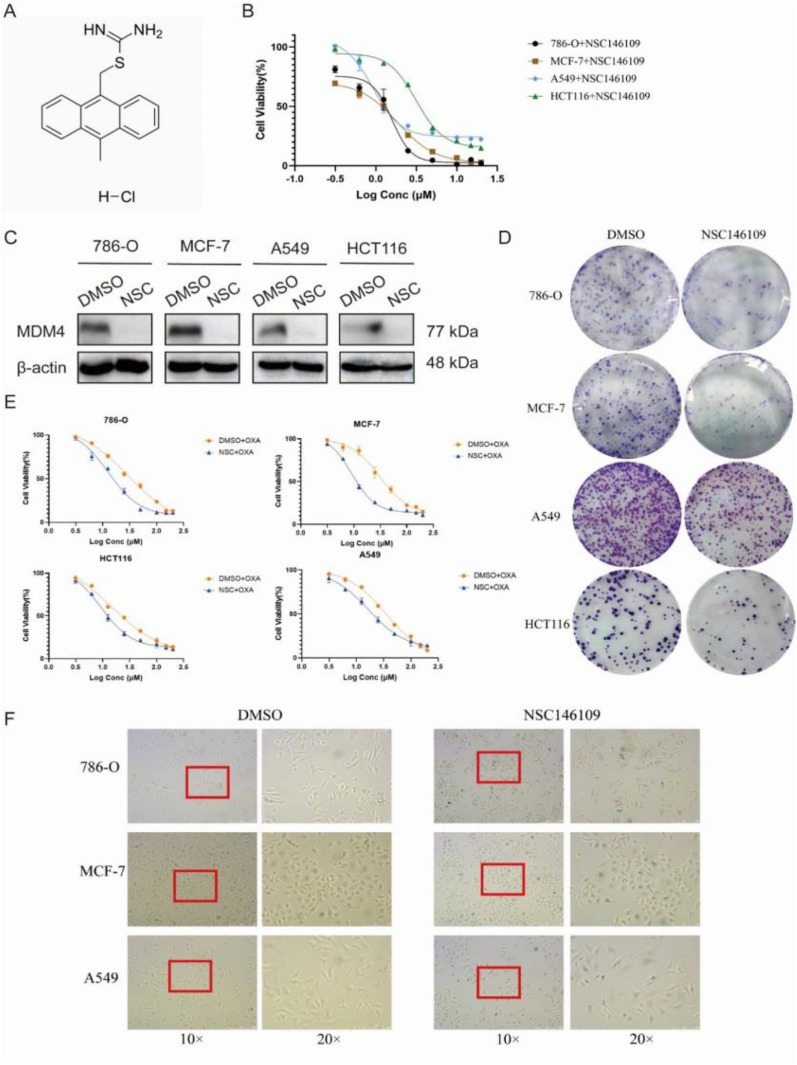
Antitumor efficacy of NSC146109 in kidney, breast, lung and colon cancer cell lines. **A** Chemical structure formula of NSC146109. **B** The 50% inhibitory concentration (IC_50_) of NSC146109 in four cell lines. **C** Western blot bands of MDM4 protein in four cell lines. **D** Colony formation assay after treatment with NSC146109. **E** Cell viability following oxaliplatin exposure detected by CCK8 assay. **F** Senescence-associated β-galactosidase activity assay after treatment with NSC146109. NSC, NSC146109; OXA, oxaliplatin

To further validate the therapeutic potential of MDM4, we conducted study on the small molecule MDM4 inhibitor, NSC146109, in different tumor cell lines (kidney cancer: 786-O; breast cancer: MCF-7; lung cancer: A549; colon cancer: HCT116). NSC146109 is a pseudourea derivative and its molecular formula is C_17_H_17_CIN_2_S (Fig. 8A). We first determined the IC_50_ value in four cell lines to evaluate the concentration used in subsequent experiments. The IC_50_ of NSC146109 in 786-O is 1.340 μM, and 1.187 μM in MCF-7, 1.170 μM in A549, 4.277 μM in HCT116 (Fig. 8B). Next, the western blot clearly suggested that the MDM4 protein expression was strongly abrogated by NSC146109 (Fig. 8C, Additional file 1: Fig. S5). Colony formation experiment was conducted to verify the impact of low MDM4 expression on the growth and proliferation potential. Although the cell proliferation rates differed in four tumor types, they were all significantly inhibited by NSC146109 (Fig. 8D). After that, we further explored whether it could increase the sensitivity to chemotherapy agents in cancer cells. As shown in Fig. 8E, there was a significant decrease in oxaliplatin IC_50_ in NSC146109-treated groups compared to DMSO-treated groups. It has been recently shown that MDM2 inhibitor can induce the senescence by activation of p53 [21], so we speculated whether inhibition of MDM4 expression would behave in the same way. Finally, we assayed the SA-β-galactosidase activity, and the results indicates that 786-O, MCF-7, and A549 presented different degrees of senescence response after treatment with NSC146109 (Fig. 8F). All the results above demonstrate targeting MDM4 offers a promising approach for antineoplastic therapy.

## Discussion

*MDM4* is indispensable for extensive life activities regulated by p53. Its germline missense mutation may cause dyskeratosis congenita and concomitant decreased telomere length or bone marrow failure [22]. *MDM4*-knockout in mice aggravates embryonic lethality mediated by p53, which might be rescued by p53 loss. However, *MDM4* amplification is often viewed as the markers of malignancy such as retinoblastomas. Through analysis of 1015 primary and 358 metastatic melanomas, Taylor E Aronff et al. [23] found that amplifications of *MDM4* are associated with higher rate of metastasis, poorer therapeutic efficacy of immunotherapy and lower overall survival in metastatic melanomas. MDM4 protein can also disrupt double-strand DNA break repair and inhibit the DNA damage response signaling pathway, resulting in genome instability [24]. This explains correlations between MDM4 and oncogenic mutations above.

As the tumor-driving proteins, MDM4 and MDM2 often exert synergistic effects in a p53-dependent manner. MDM4 and MDM2 protein can bind to p53 protein through protein–protein interactions to prevent its expression under stress conditions and then inactivate or weaken its transcriptional function. The C-terminal of MDM2 is a Ring domain with E3 ubiquitin ligase activity, which can ubiquitinate p53, thereby causing its degradation and loss of function. Although MDM4 and MDM2 have a high degree of similarity, the Ring domain of MDM4 has no E3 ubiquitin ligase function to degrade p53 [25]. However, in vitro studies have shown that MDM4 mainly attenuates p53 transcriptional activity instead of its protein stabilization. There is evidence that MDM4 interacts with topoisomerase IIα (TOP2A) and thereby enhances the repressive effect on p53 expression [26]. On the other hand, MDM4 directly binds to MDM2 and promotes its regulatory function of p53. The study by Jing Yang et al. [27] reported that MDM4 potentiates MDM2 E3 ligase function through the recruitment of UbcH5c and brings about p53 degradation in vivo. Moreover, MDM4 is not just limited to the regulation of p53. It could independently modulate DNA maintenance, lipid storage, and estrogen signaling [28]. Venkatesh et al. believed that MDM4–MDM2 complex disrupts lipid metabolism to favor ferroptosis by increasing PPARα activity [29]. In our research, we found that high MDM4 expression significantly activates Wnt, MAPK, JAK-STAT, TGF-β or some other oncogenic signaling pathways. Consistently, previous study had shown that MDM4 is a physiological inhibitor of CK1α and disruption of MDM4-CK1α interaction in vivo reduces Wnt/β-catenin pathway expression [30]. This phenomenon has been observed even in acute myeloid leukemia [31]. Recent study differs from our research in that the wild-type p53 cell line was selected to investigate the regulation of transcriptomics by MDM4 in primary uveal melanoma [32]. The results showed that cell cycle regulatory genes were repressed and cell death activating genes were stimulated upon MDM4 depletion. Knockdown of MDM4 had a less inhibitory effect on growth in the presence of FOXO depletion. This reveals that MDM4 appeared to act as an oncogene by regulating FOXO tumor suppressor function.

With the development of medical technology, immunotherapy has become the fourth treatment method for cancer, only after surgery, chemotherapy and radiotherapy [33]. The immune system plays an essential role in the recognition and elimination of foreign entities, construction of a vital defense mechanism, and maintenance of internal homeostasis [34]. It has been shown that MDM4–Ser314 phosphorylation-mediated p53 regulation can promote M1 polarization in macrophages, thereby generating an immunosuppressive microenvironment that promotes tumor progression [35]. Our findings also validate some transcriptional changes of immune-related molecules when MDM4 is overexpressed. DDX58 belongs to a family of cytosolic pattern recognition receptors (PRR), which can detect viral RNA followed by facilitating an innate immune response [36]. It regulates IRF3-, IRF-dependent expression of type 1 and type III interferons and the NF-κB-dependent expression of proinflammatory cytokines [37]. TNFSF18 protein, GITRL, is a specific ligand for the tumor necrosis factor (TNF) receptor family-related protein [38]. Once they are activated, they stimulate each other and activate intracellular signals to regulate immune function. CHST4 encodes an N-acetylglucosamine 6-O sulfotransferase involved in lymphocyte trafficking and homing [39]. Zhang et al. [40] found that CHST4 expression is associated with immune cell infiltration such as B cells, CD4 + T cells, macrophages, dendritic cells, and neutrophils. The mechanisms underlying these changes are worth further in-depth study.

Because of the remarkable carcinogenic properties of MDM4, many peptide-based and small-molecule inhibitors of MDM4 have been developed, including NSC146109 (XI-011), NSC207895 (XI-006), RO-5963, SJ-172550 and so on. A 12-residue peptide (pDI) was discovered as MDM4 inhibitor, mainly due to the cocrystal structure of pDI/MDM4 displaying identical binding sites to the p53 peptide on three key hydrophobic residues [41]. NSC146109 (XI-011), one of small-molecule MDM4 inhibitors, was proven effective in liver, breast, cervical, head and neck cancer [42–45]. In concordance with our experimental results, it inhibits cell proliferation, suppresses tumor cell growth and enhances the cytotoxicity of cisplatin in vivo and in vitro. Among those, MDM4 depletion is thought to augment gemcitabine sensitivity by impacting DNA damage/repair in a p53-independent fashion [46]. As for the mechanism of triggering cellular senescence, it is well-reported that the knockout of MDM4 markedly increased the key regulator, p27, even in p53-mutant human prostate cancer cells [47]. Hence, further studies are needed to figure out whether MDM4 works through direct or indirect effects. In addition to selective MDM4 inhibitor, there is dual MDM2/MDM4 inhibitor that binds to MDM4 with weaker affinity than MDM2. Such dual inhibitors have tended to focus on p53/MDM2/MDM4 interactions instead of MDM4 per se. ALRN-6942, currently the only drug now in early-phase clinical trials, exhibits the activation of wild-type p53 following by dual inhibition of MDM2 and MDM4 [48]. It may contribute to specific targeted therapies for myeloid neoplasms and leukemia with low side effects [49, 50], because the pharmacological effect on cell-cycle arrest effectively reduces therapeutic toxicity in treatment cycles. Recently, targeting ubiquitin specific protease 7 (USP7) was proposed as a novel treatment strategy, and the reason is that USP7 protects MDMX from ubiquitination-mediated proteasomal degradation [51]. In short, studies on MDM4 inhibitors drive the R&D of new targeted drugs.

In this work, we did not further explore the different expression genes obtained from transcriptomic sequencing, nor did we delve into the pathways by which MDM4 affects tumor cell immune infiltration. Given the multi-functional nature of MDM4, the detailed mechanism and involved pathway of MDM4 in tumor needed further investigation.

As a whole, *MDM4* is universally upregulated in a raft of tumors, and its overexpression correlates with the pathological staging and clinical prognosis of patients with different kinds of tumors. *MDM4* expression positively correlates with TMB, HRD, MSI, LOH and the score of immune cell, and its effect on tumor immune function varies according to tumor type. The small-molecule inhibitor of MDM4, NSC146109, exhibits the tumor-suppressive activity in multiple cancer cell lines such as breast, kidney, lung and colon cancer. Our study is the first attempt to perform a pan-cancer analyses of MDM4, which confirms the importance of MDM4 in tumor diagnosis, helps to explore its mechanism in tumor development, and provides better options for successful implementation of targeted therapies.

### Supplementary Information

**Additional file 1**. **Table 1.** Primer sequences for qPCR experiment. **Figure 1.** Diagnostic Effectiveness of MDM4. (A, B, C, D) Receiver Operating Characteristic Curve analysis for the expression levels of MDM4 in HNSC, KICH KIRC and READ. Correlation between the MDM4 expression and clinical stages of HNSC (E), pathologic stage of THCA (F), WHO grade of GBMLGG (G), histologic grades of LIHC(H), and histological type of ESCA(I). **Figure 2.** Kaplan-Meier survival curves of MDM4 in cancers. Kaplan-Meier survival curves for overall survival in PCPG (A), PRAD (B) and THYM (C). Kaplan-Meier survival curves for disease specific survival in ACC (D), PAAD (E), and PRAD (F). Kaplan-Meier survival curves for progress free interval in GBMLGG (G), KICH (H) and READ (I). **Figure 3.** Mutation waterfall plot of MDM4. Mutation waterfall plot between the high vs. low MDM4 expression groups in COAD(A), STAD(B) and PRAD(C). **Figure 4.** Heat map showing MDM4 immune infiltration level. Heat map showing the level of immune cell infiltration with IPS (A) and MCPcounter (B) algorithm. **Figure 5.** Detection of MDM4 protein in Huh7 and SW480 cell lines treated with/without MDM4 inhibitor NSC146109 using Western-blot.

## Data Availability

This paper involves all data come From the UCSC database (https://xenabrowser.net/), Cancer Cell Line Encyclopedia database (https://sites.broadinstitute.org/ccle), Human Protein Atlas (https://www.proteinatlas.org/), TCGA database (https://portal.gdc.cancer.gov/) and GDC (https://portal.gdc.cancer.gov/). The RNA-seq data from this study have been deposited to the GEO database (https://www.ncbi.nlm.nih.gov/geo/query/acc.cgi?acc=GSE243108) and assigned the identifier GSE243108.
