# Supplementary material for: MDM4 was associated with poor prognosis and tumor-immune infiltration of cancers
Source: Eur J Med Res. 2024 Jan 27;29:79. doi: 10.1186/s40001-024-01684-z (PMC10821240; doi:10.1186/s40001-024-01684-z)
Supplement: Supplementary file 1 — Additional file 1. Table 1. Primer sequences for qPCR experiment. Figure 1. Diagnostic Effectiveness of MDM4. (A, B, C, D) Receiver Operating Characteristic Curve analysis for the expression levels of MDM4 in HNSC, KICH KIRC and READ. Correlation between the MDM4 expression and clinical stages of HNSC (E), pathologic stage of THCA (F), WHO grade of GBMLGG (G), histologic grades of LIHC(H), and histological type of ESCA(I). Figure 2. Kaplan-Meier survival curves of MDM4 in cancers. Kaplan-Meier survival curves for overall survival in PCPG (A), PRAD (B) and THYM (C). Kaplan-Meier survival curves for disease specific survival in ACC (D), PAAD (E), and PRAD (F). Kaplan-Meier survival curves for progress free interval in GBMLGG (G), KICH (H) and READ (I). Figure 3. Mutation waterfall plot of MDM4. Mutation waterfall plot between the high vs. low MDM4 expression groups in COAD(A), STAD(B) and PRAD(C). Figure 4. Heat map showing MDM4 immune infiltration level. Heat map showing the level of immune cell infiltration with IPS (A) and MCPcounter (B) algorithm. Figure 5. Detection of MDM4 protein in Huh7 and SW480 cell lines treated with/without MDM4 inhibitor NSC146109 using Western-blot. [file 40001_2024_1684_MOESM1_ESM.docx]

**Supplementary Table 1. Primer sequences for qPCR experiment**.

| Gene | NCBI Accession number | Forward/Reverse | Sequence |
| --- | --- | --- | --- |
| CHST4  (human) | NM_001166395.2 | forward | 5’- AGGAGGTGCGCTTCTTCAAC-3’ |
|  |  | reverse | 5’- AGGGTTGGTCCTCCTTCTTG-3’ |
| TNFSF18  (human) | NM_005092.4 | forward | 5’- ACCCTCAAAATGGCAAATGGC-3’ |
|  |  | reverse | 5’- GTAGTTTGCATTGGGAGCCAC-3’ |
| DDX58  (human) | NM_001385910.1 | forward | 5’-GCTGATGAAGGCATTGACATTG-3’ |
|  |  | reverse | 5’- CACTTGCTACCTCTTGCTCTT-3’ |
| OAS1  (human) | NM_001406022.1 | forward | 5’- ACTGGCGGCTATA AACCTAACC-3’ |
|  |  | reverse | 5’- TTGACTAGGCGGATGAGGCT-3’ |
| XAF1  (human) | NM_001353137.2 | forward | 5’- GGGTAAATGTTGTCCAGACTCAG-3’ |
|  |  | reverse | 5’- GCTTCTTGGTGCTTTCTTTGATG-3’ |
| IFI27  (human) | NM_001288952.2 | forward | 5’- AGCCTTGTGGCTACTCTGC-3’ |
|  |  | reverse | 5’- GTAGAACCTCGCAAT GACAGC-3’ |
| IFI44  (human) | NM_006417.5 | forward | 5’-AGTTGGTAAACGCTGGTGTGG-3’ |
|  |  | reverse | 5’- GGACTTCCTCTAGCTTGGACCT-3’ |
| IFIT3  (human) | NM_001031683.4 | forward | 5’- GCTGAGTCCTGATAACCAATACG-3’ |
|  |  | reverse | 5’- TTCCAAGGCTTCTTCAACAAACT-3’ |
| GAPDH  (human) | NM_001357943.2 | forward | 5’- GGTGTGAACCAT GAGAAGTATGA-3’ |
|  |  | reverse | 5’- GAGTCCTTCCACGATACCAAAG-3’ |
| iNOS  (human) | NM_031252.2 | forward | 5’- TCACCTACTTCCTGGACATCAC-3’ |
|  |  | reverse | 5’- GAACTTCCACTTGCTGTACTCTG-3’ |
| CD80  (human) | NM_001314054.1 | forward | 5’- GGAATACAACCAAGCAAGAGCAT-3’ |
|  |  | reverse | 5’- TAGGTCAGGCAGCATATCACAA-3’ |
| STAT6  (human) | NM_001311083.1 | forward | 5’- GAGCCACTACAAGCCTGAAC-3’ |
|  |  | reverse | 5’- GCCATTCCAAGGTCATAAGAAGG-3’ |
| ARG1  (human) | NM_001037859.2 | forward | 5’- GGAAGACACCAGAAGAAGTAACTC-3’ |
|  |  | reverse | 5’- GGTTAAGGTAGTCAATAGGCTTGT-3’ |
| MDM4  (human) | NM_001204172.2 | forward | 5’- TGATTGTCGAAGAACCATTTCGG-3’ |
|  |  | reverse | 5’- TGCAGGGATCAAAAAGTTTGGAG-3’ |


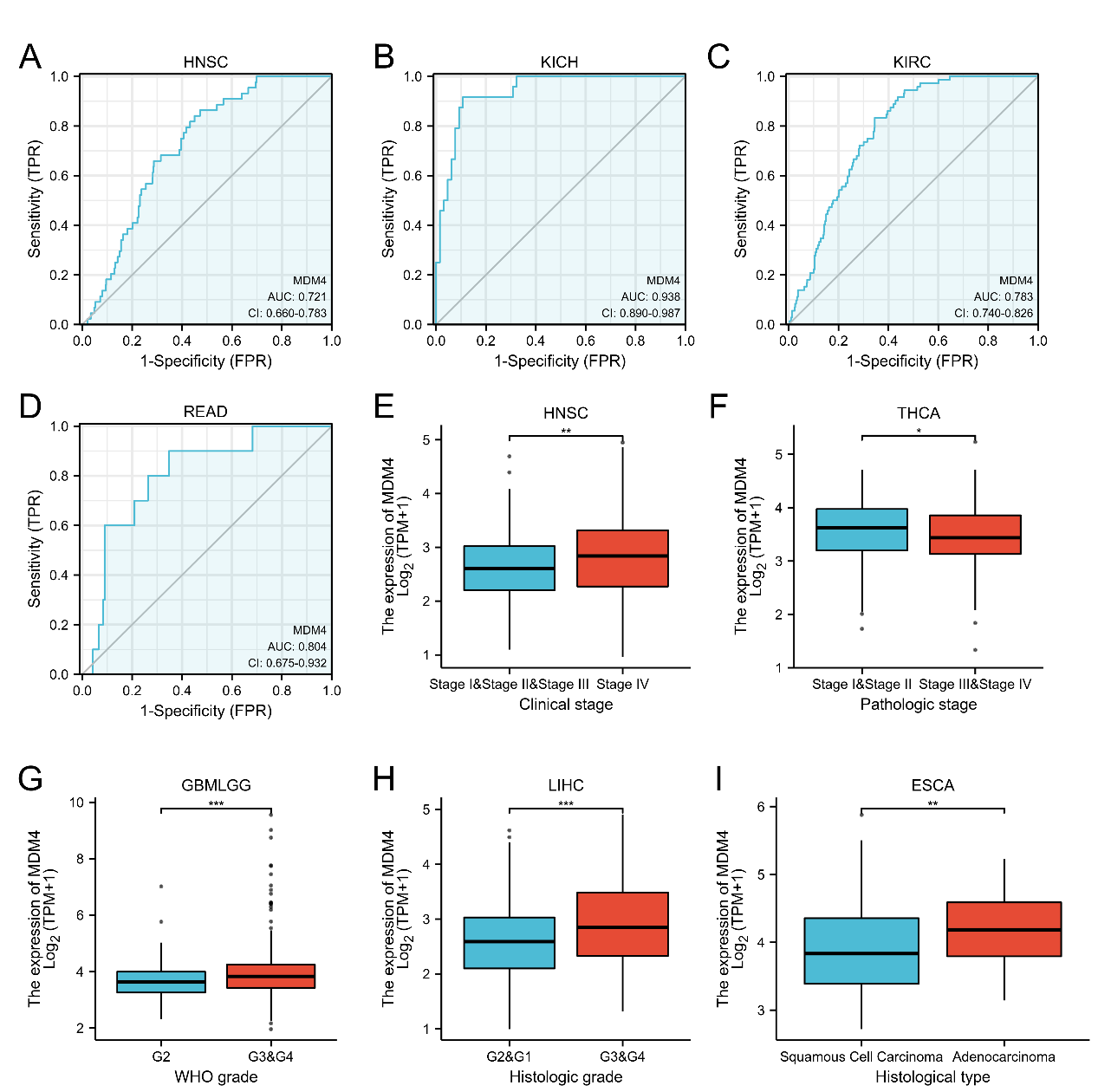


**Supplementary Figure 1. Diagnostic Effectiveness of MDM4.** (A, B, C, D) Receiver Operating Characteristic Curve analysis for the expression levels of MDM4 in HNSC, KICH KIRC and READ. Correlation between the MDM4 expression and clinical stages of HNSC (E), pathologic stage of THCA (F), WHO grade of GBMLGG (G), histologic grades of LIHC(H), and histological type of ESCA(I).


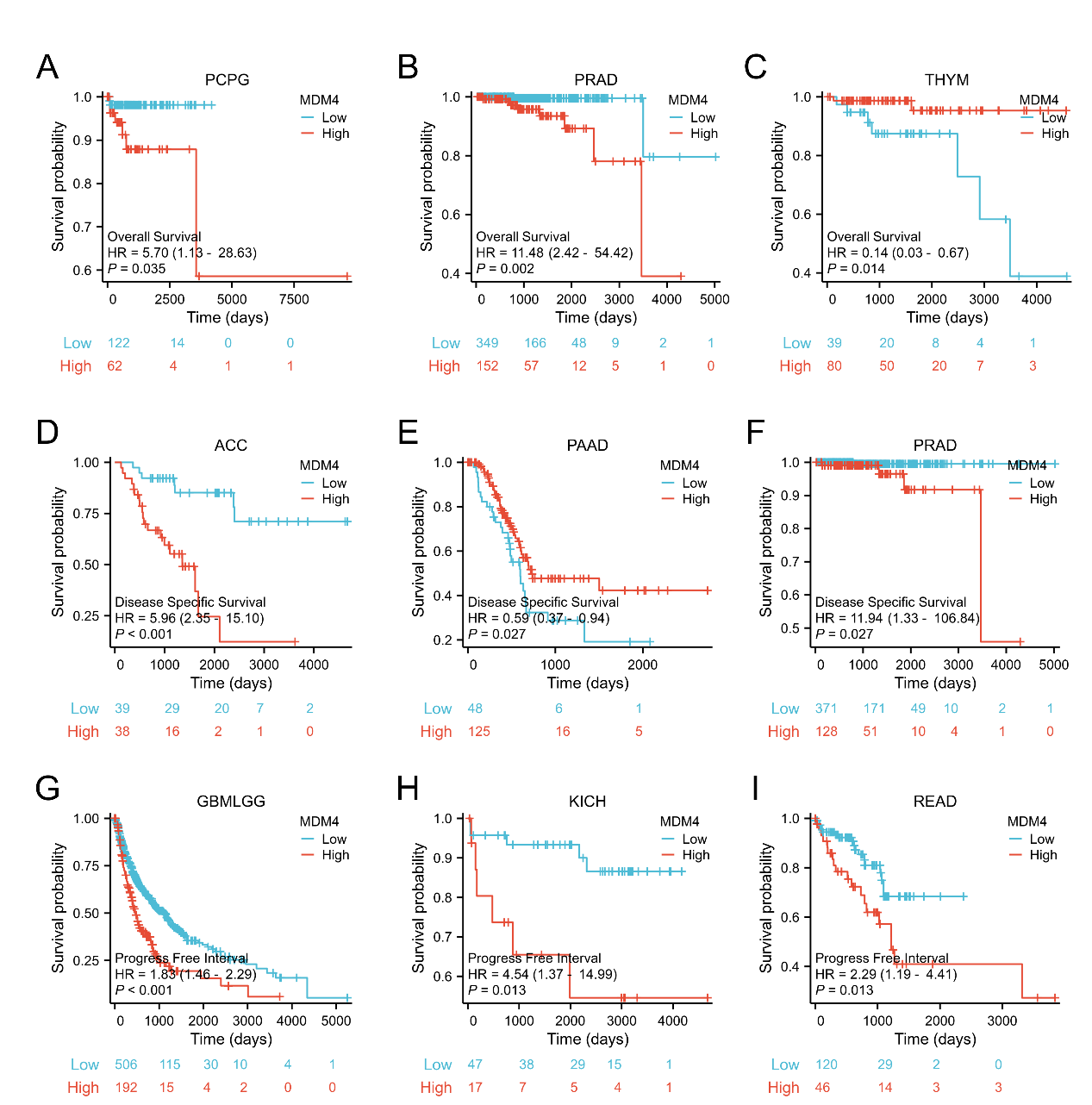


**Supplementary Figure 2. Kaplan-Meier survival curves of MDM4 in cancers.** Kaplan-Meier survival curves for overall survival in PCPG (A), PRAD (B) and THYM (C). Kaplan-Meier survival curves for disease specific survival in ACC (D), PAAD (E), and PRAD (F). Kaplan-Meier survival curves for progress free interval in GBMLGG (G), KICH (H) and READ (I).


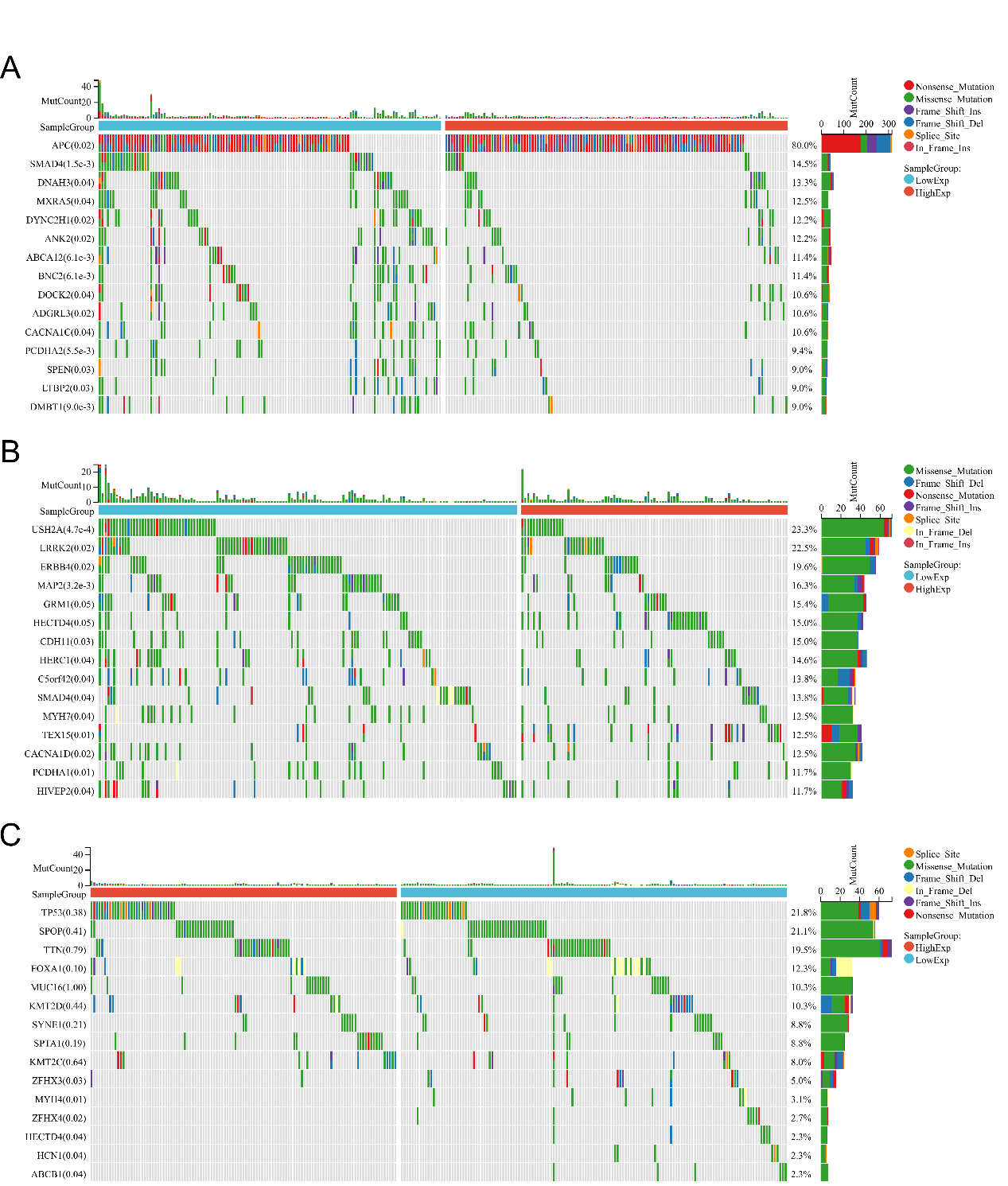


**Supplementary Figure 3. Mutation waterfall plot of MDM4.** Mutation waterfall plot between the high vs. low MDM4 expression groups in COAD(A), STAD(B) and PRAD(C).


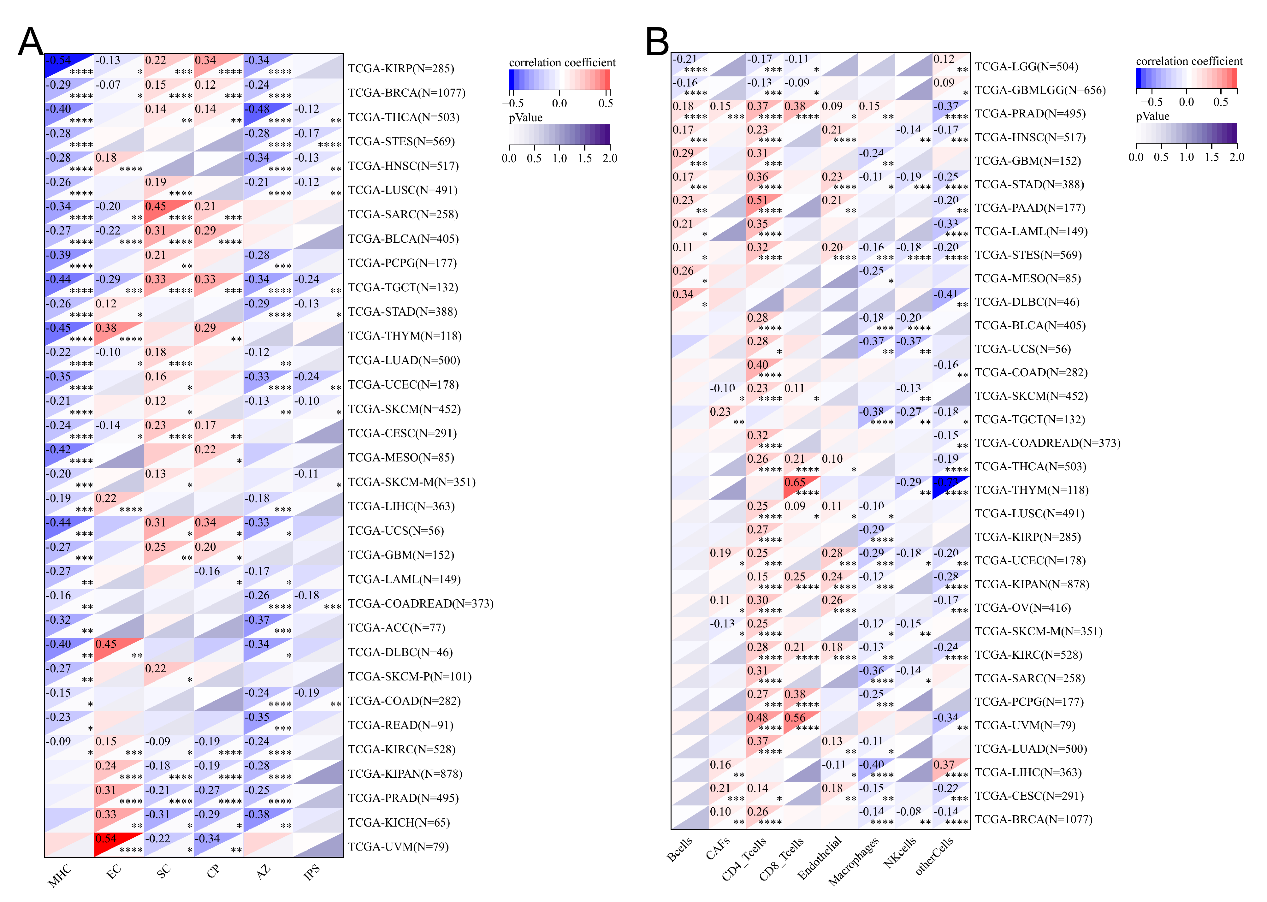


**Supplementary Figure 4.** **Heat map showing MDM4 immune infiltration level.** Heat map showing the level of immune cell infiltration with IPS **(A)** and MCPcounter **(B)** algorithm.


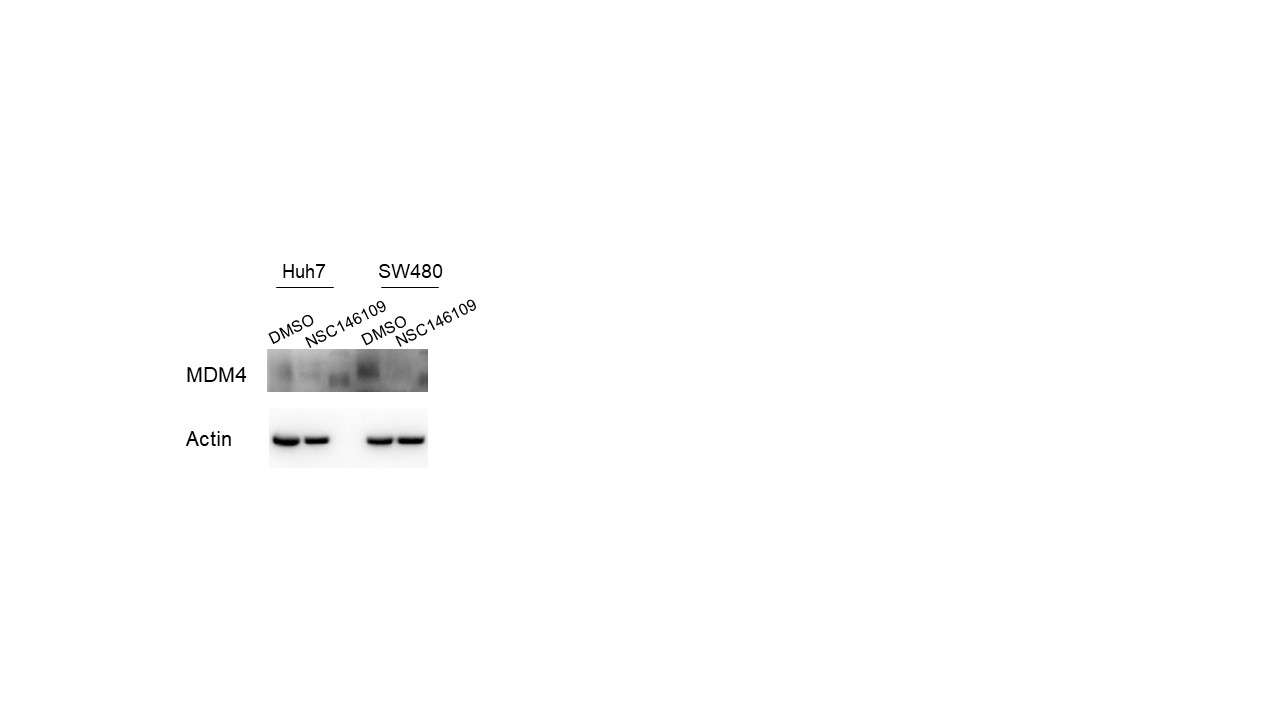


**Supplementary Figure 5. Detection of MDM4 protein in Huh7 and SW480 cell lines treated with/without MDM4 inhibitor NSC146109 using Western-blot.**
